# [Retracted] Long non-coding RNA HOTAIR modulates HLA-G expression by absorbing miR-148a in human cervical cancer

**DOI:** 10.3892/ijo.2024.5685

**Published:** 2024-09-02

**Authors:** Jinbao Sun, Haipeng Chu, Jianghai Ji, Gaoxiang Huo, Qinglei Song, Xue Zhang

Int J Oncol 49: 943-952, 2016; DOI: 10.3892/ijo.2016.3589

Following the publication of this paper, it was drawn to the Editors' attention by a concerned reader that the HLA western blotting data shown for the HeLa cell line in Fig. 3D on p. 948 were strikingly similar to data appearing in different form in Fig. 3 in the following article written by different authors at different research institutes that was submitted for publication at around the same time, and for which an Expression of Concern has subsequently been published: Sun L, Xue H, Jiang C, Zhou H, Gu L, Liu Y, Xu C and Xu Q: LncRNA DQ786243 contributes to proliferation and metastasis of colorectal cancer both *in vitro* and *in vivo*. Biosci Rep 36: e00328, 2016. In addition, it was also noted that certain of the control western blotting data featured in Figs. 3D and 5B were strikingly similar, even though different experiments were being reported on in these figures.

In view of the fact that the contentious data were submitted for publication at around the same time, the Editor of *International Journal of Oncology* has decided that this paper should be retracted from the Journal. The authors were asked for an explanation to account for these concerns, but the Editorial Office did not receive a reply. The Editor apologizes to the readership for any inconvenience caused.

